# Repetitive Transcranial Magnetic Stimulation Improves Neurological Function and Promotes the Anti-inflammatory Polarization of Microglia in Ischemic Rats

**DOI:** 10.3389/fncel.2022.878345

**Published:** 2022-04-12

**Authors:** Jing Luo, Yuan Feng, Mingyue Li, Mingyu Yin, Feng Qin, Xiquan Hu

**Affiliations:** ^1^Department of Rehabilitation Medicine, The Third Affiliated Hospital, Sun Yat-sen University, Guangzhou, China; ^2^Department of Hepatobiliary Surgery, The Third Affiliated Hospital, Sun Yat-sen University, Guangzhou, China; ^3^Department of Neurosurgery, Lingnan Hospital, The Third Affiliated Hospital, Sun Yat-sen University, Guangzhou, China

**Keywords:** rehabilitation, repetitive transcranial magnetic stimulation, ischemic stroke, inflammation, microglia

## Abstract

Ischemic stroke (IS) is a severe neurological disease that is difficult to recovery. Previous studies have shown that repetitive transcranial magnetic stimulation (rTMS) is a promising therapeutic approach, while the exact therapy mechanisms of rTMS in improving neural functional recovery remain unclear. Furthermore, the inflammatory environment may influence the rehabilitation efficacy. Our study shows that long-term rTMS stimulation will significantly promote neurogenesis, inhibit apoptosis, and control inflammation. rTMS inhibits the activation of transcription factors nuclear factor kappa b (NF-κB) and signal transducer and activator of transcription 6 (STAT6) and promotes the anti-inflammatory polarization of microglia. Obvious promotion of anti-inflammatory cytokines production is observed both *in vitro* and *in vivo* through rTMS stimulation on microglia. In addition, neural stem cells (NSCs) cultured in conditioned medium (CM) from microglia treated with rTMS showed downregulation of apoptosis and upregulation of neuronal differentiation. Overall, our results illustrate that rTMS can modulate microglia with anti-inflammatory polarization variation, promote neurogenesis, and improve neural function recovery.

## Introduction

Ischemic stroke (IS) acts as a leading cause of death and disability (Benjamin et al., 2019; Kelmanson et al., 2021). Due to the limitation of the treatment time window and medical conditions, about 70–80% of the surviving patients with cerebral infarction still have varying degrees of dysfunction (Girotra et al., 2020). Apoptosis and necrosis are the two main cell death modes in the acute phase of cerebral infarction (Lipton, 1999), and neurogenesis is an important basis for the recovery of nerve function (Koh and Park, 2017). Therefore, how to inhibit nerve apoptosis and promote neurogenesis is a momentous research direction for the recovery of nerve function after cerebral infarction.

The rapid development of rehabilitation medicine makes it possible to restore the neurological function of stroke patients well and greatly improves the quality of life. Repetitive transcranial magnetic stimulation (rTMS), a non-invasive brain stimulation technology based on the principle of Faraday electromagnetic induction (Vidal-Dourado et al., 2014), can effectively promote the recovery of neurological function. Based on the induction of repetitive current pulses in the brain, rTMS is obtained by applying an electromagnetic coil to generate a magnetic field near the scalp. For the advantages in terms of non-invasiveness and safety, with minimal or no side effects (Peruzzotti-Jametti et al., 2013), rTMS has been widely used in clinical rehabilitation treatment of patients with cerebral infarction (Luber and Lisanby, 2014; Koch et al., 2019; Liu et al., 2020; Wang et al., 2020; Yin et al., 2020). Relative studies believe that rTMS acts on neurorehabilitation and may relate to the following factors: mutual inhibition between the bilateral hemispheres of the brain (Carson, 2020), regulating the excitability of the brain (Hallett, 2000; Tan et al., 2013), improving brain metabolism and cerebral blood flow (Paillere et al., 2011; Shinba et al., 2018), and rebuilding brain function networks and regulating the transmission of a variety of neurotransmitters (Yin et al., 2020; Natale et al., 2021). Previous reports by us and others confirmed that rTMS could improve the neurological function after cerebral infarction and promote neurogenesis (Guo et al., 2017; Luo et al., 2017; Cui et al., 2019). It can be seen that rTMS has a very wide range of functions, and its internal mechanism is also quite complicated which needs to be further elucidated.

Many strategies have been trialed to find the mechanism for obtaining the best effect of rTMS treatment. For such purposes, many studies focus on the mechanism of rTMS acting on the neural stem cells (NSCs) and neural precursor cells (NPCs), which are related to neurogenesis, and astrocytes or microglia connected with neuroinflammation (Hong et al., 2020). For instance, rTMS has therapeutic effects on depression because it can reverse the decrease of NSCs and enhance neurogenesis, and rTMS could regulate the balance between proliferation and apoptosis of NSCs (Zhao et al., 2019). For neuroprotective effect, it has been reported that rTMS could improve functional recovery by inhibiting neurotoxic polarization of astrocytes in ischemic rats, which means rTMS may regulate inflammation through the action of neural glial cells (Sasso et al., 2016; Cullen et al., 2019; Clarke et al., 2021).

Microglia, one of the macrophages residing in the central nervous system, plays a key role during neural damage (Nayak et al., 2014; Prinz et al., 2019). Traditionally, microglia activation has been thought to play a detrimental role in IS, for its inhibition can reduce brain damage caused by ischemia stroke (Ma et al., 2017). However, a growing number of evidences indicate it is essential for promoting nerve recovery after stroke, and the differential polarization of microglia may explain the biphasic effect (Xiong et al., 2016; Kanazawa et al., 2017; Ma et al., 2017). Many studies show that rTMS can reduce the release of proinflammatory cytokines and inhibit the activation of microglia (Kim et al., 2013; Yang et al., 2020; Zong et al., 2020), whereas contradictory research indicates that rTMS could induce the microglia to inflammatory phenotype, while reducing neurogenesis (Muri et al., 2020).

At present, the function of rTMS on microglia of IS is still unclear. The aim of this study was to explore the changes in the phenotype and function of microglia after rTMS treatment. At the same time, we also observed the effects of rTMS-stimulated microglia supernatant on the proliferation and apoptosis of NSCs.

## Materials and Methods

### Rats

A total of 80 male spontaneously hypertensive rats (SHRs) weighing 200–240 g were purchased from the Beijing Vital River Laboratory Animal Technology Co. Ltd., China. SHRs were allocated to each group randomly. Rats were housed in the same animal care facility during a 12 h light/dark cycle throughout the protocol with free access to food and water. All animal protocols were approved and reviewed by the Sun Yat-sen University Institutional Animal Care and Use Committee.

### Transient Middle Cerebral Artery Occlusion

Middle cerebral artery occlusion (MCAO) surgery was performed on SHR under anesthesia as our prior studies described (Luo et al., 2017). In brief, SHRs were anesthetized by intraperitoneal injection of 3.5% chloral hydrate (350 mg/kg). A filament (nylon suture with a rounded tip) was inserted into the left internal carotid artery to block the left middle cerebral artery. After 90 min of MCAO, the filament was pulled back to restore blood flow (reperfusion). Criteria for successful model preparation were that after the rat was awake from anesthesia, the left limb was paralyzed, and the modified neurological severity score (mNSS) score met the inclusion. Rats with moderately neurological dysfunction with a mNSS of 7–12 were included in the group. Exclusion criterion included rats that were found to have subarachnoid hemorrhage or no paralysis, or died before the sampling time, mild (mNSS of 1–6) or severely damaged (mNSS of 13–18). For the proliferation assay, daily injections of bromodeoxyuridine (BrdU, 50 mg/kg; B5002, Sigma-Aldrich, United States) were given intraperitoneally for every group at 1 day after the MCAO, to label dividing cells until 7 days after MCAO.

### Isolation and Culture of Rat Microglia

Microglia were isolated from cultures of newborn Wistar rat brain and tissue dissociated by trituration with trypsin (Gibco, United States) as described previously (Giulian and Baker, 1986). In brief, isolated cerebral cortices from newborn Wistar rats were taken off the meninges, minced in Roswell Park Memorial Institute (RPMI) 1640 basal medium (Gibco, United States), a complete medium and 10% fetal bovine serum (FBS, Gibco, United States) before being dissociated by trituration in 0.25% trypsin. Cells were plated in 75 cm^2^ plastic culture flasks containing 10 ml complete medium with 10% FBS at a density of 1 × 10^6^ cells/ml. Culture flasks were vigorously agitated on a rotary shaker for 12 h (37°C, 180 rpm) after 7 days of culture. Then, GFAP (+) astroglia remained adherent to the flasks, and the resulting cell suspension, rich in microglia, was placed in new plastic flasks (10^5^ cells/ml) and allowed to adhere at 37°C.

### Isolation and Culture of Rat Neural Stem Cells

Neural stem cells were isolated from fetal rats at embryonic day 14.5 (E14.5) as previously reported (Azari et al., 2011). Briefly, brains from embryos were isolated and dissociated by accutase (Gibco, United States). Then, cell suspension was filtered with a 70 μm cell strainer. Ammonium chloride lysis was used to remove red blood cells, and the rest cells were added to culture flasks in NSCs culture medium. NSCs culture medium contained low glucose-Dulbecco’s Modified Eagle’s Medium (L-DMEM) with 2% (v/v) B27 (Gibco, United States), epidermal growth factor (EGF, 20 ng/mL, PeproTech) and mitogens basic fibroblast growth factor (bFGF, 20 ng/mL, PeproTech).

### Experimental Grouping and Repetitive Transcranial Magnetic Stimulation

*In vitro*, microglia were randomly divided into tms (+) group and TMS (−) group. In TMS (−) group, 1 μg/ml lipopolysaccharide (LPS) was added in microglia culture medium to activate microglia for 18 h. Then, the medium was replaced with fresh medium and continued to culture for 72 h before subsequent testing. In TMS (+) group, microglia were activated by LPS as TMS (−) group did. Then, microglia in fresh culture medium were treated with rTMS one time a day for 2 days. Microglia in the TMS (+) group were applied at 10 Hz, 30% maximum output intensity of the machine, with 20 pulses per train, 10 s intertrain interval, and a of total 60 trains (1,200 pulses) for 11 min 44 s. Microglia were used for subsequent testing after 72 h culture (rTMS were treated two times). Microglial-derived conditioned medium (CM) from above two groups were collected, centrifuged, and stored at −80°C for subsequent experiments.

*In vivo*, SHRs were randomly divided into three groups, namely, SHAM group (*n* = 24), TMS (−) group (*n* = 24), and TMS (+) group (*n* = 24). All groups were divided into two subgroups on the 7th and 28th days after rTMS (*n* = 12). Rats in the TMS (+) group received 10 Hz rTMS with a total of 60 trains, 20 pulses per train (1,200 pulses), 10 s intertrain interval, for 11 min 44 s. Rats in the TMS (−) group experienced the same experimental manipulations by placing an inactive coil on the rats’ head. SHR receiving rTMS were stimulated for 7 days (short-term observation point) and 28 days (long-term observation point) during a 2-day period a week, beginning at 2 days after MCAO. The SHAM group were housed in standard cages supplied with adequate food and water but received no stimulation.

TMS treatment was conducted with a MagPro X100 magnetic stimulators (The MagVenture Company, Denmark). The magnetic stimulation coil is an ultra-small figure-of-eight coil (MC-B35, Dantec, Denmark) specially designed for animals, with 24 mm inner diameter and 47 mm outer diameter, an output frequency of 1–100 Hz, and a maximum output magnetic field strength of 4.2 T. Motor-evoked potentials (MEPs) were measured at the right hind limbs, quadriceps femoris muscle, using electromyography (MedelecSynergy; Oxford Instruments, Surrey, United Kingdom), as previously described (Luo et al., 2017).

### Primary Microglia Proliferation Assay

Carboxyfluorescein succinimidyl ester (CFSE, Invitrogen, United States) staining (2 μmol/L) was used to assess primary microglia (PM) proliferation. The cells were incubated for 10 min at 37°C. Then, the original staining volume of culture medium were added five times to the cells and incubated for 5 min. The cells were pelleted by centrifugation and then suspended and distributed to 6-well plates. After 3 days of culture, microglia were collected and analyzed by flow cytometry.

### Neural Stem Cells Assay

Neural stem cells were dissociated and cultured in matrigel-coated (Corning, United States) 6-wells culture plates at a density of 2 × 10^5^/well. Cells were cultured with NSCs culture medium for 48 h. Then, the culture medium was replaced by CM collected from microglia to promote differentiation. Culture medium were changed every 3 days.

For the proliferation assay, NSCs were tested at day 3 after cultured with microglia CM. NSCs were incubated with 10 mM BrdU at 37°C for 6 h. Then, cells were fixed with paraformaldehyde (PFA) and incubated in 2 mol/L HCl and washed in 1 M borate solution two times. Later, cells were blocked with 5% normal goat serum for 1 h at room temperature. Then, cells were incubated with BrdU (ab207175, Abcam, United States) and NESTIN (ab81462, Abcam, United States) antibodies overnight at 4°C. Finally, cells were incubated with secondary antibody and Hoechst stain before examined with the fluorescence microscope.

For TUNEL staining, NSCs were tested at day 3 after cultured with microglia CM. NSCs were stained with the TUNEL technique using an apoptosis detection kit (MA0223, Meilunbio, China) according to the manufacturer’s protocols. In brief, cells were fixed with fix solution (MA0192, Meilunbio, China) for 30 min. Proteinase K (20 μg/ml) was added to each sample so that the solution covers the entire sample area and incubated for 5 min at room temperature. Furthermore, 50 μl TUNEL detection solution was added dropwise to the sample and incubated in the dark for 60 min at 37°C. After all, the slices were washed 2–3 times with PBS and examined under an EVOS M5000 imaging system.

For differentiation assay, cells were collected at day 9, and RNA was isolated by RNA Quick Purification kit (ESscience, China). qPCR was used to test the expression levels of specific markers.

### Enzyme-Linked Immunosorbent Assay

Tumor necrosis factor (TNF)-α, interleukin (IL)-1β, IL-4, and IL-10 production was measured using enzyme-linked immunosorbent assay (ELISA) quantitation kits (all from MEIMIAN, China) as per the manufacturer’s protocols.

### RNA Isolation and qPCR

RNA was isolated from the brain tissue by RNA Quick Purification kit (ESscience, China). cDNA was prepared using a Revert Aid First Strand cDNA Synthesis Kit (Thermo Scientific, United States). The cDNA obtained was subjected to qPCR with the SYBR Green reagent (Thermo Scientific, United States) using the rat primers listed in Supplementary Table 1. Expression levels were normalized to those of glyceraldehyde-3-phosphate dehydrogenase (GAPDH). The primers are listed in Supplementary Table 1.

### Western Blotting Analysis

Cells and brain tissues were extracted, and the protein concentration was measured using a bicinchoninic acid (BCA) protein assay kit (Beyotime, China). Proteins were separated using 8 or 12% sulfate-polyacrylamide gel electrophoresis and then transferred to a polyvinylidene fluoride (PVDF) membrane. Then, the membrane was blocked with Tris-buffered saline (TBS)/T containing 5% non-fat dry milk and analyzed for the target proteins. The specific antibodies used recognized cleaved-caspase3, p-nuclear factor of kappa light polypeptide gene enhancer in B-cell inhibitor, alpha (IκBα) (AP0707, ABclonal, China), p-NFκB (AP0123, ABclonal, China), and p-STAT6 (#56554, CST, United States).

### Immunofluorescent Staining

For cells assay, microglia were washed with PBS and fixed with 15 min treatment of 4% cold PFA. Fixed cells were washed three times before being treated with permeabilization buffer. Furthermore, cells were treated with primary antibodies incubated in PBS overnight at 4°C. All cells were washed three times and treated with secondary antibodies for 1 h at room temperature in the next morning. Cells were then washed three times before being mounted using Fluoroshield with DAPI and glass coverslips. Cells were imaged using an EVOS M5000 imaging system (Thermo Fisher Scientific, United States).

Brain sections were incubated in 2 N HCl at 37°C for 30 min for BrdU immunostaining and washed in 0.1 M borate solution two times for 10 min, incubated in 3% H_2_O_2_ for 30 min, and blocked with 5% normal goat serum for 1 h at room temperature. BrdU (AB207175, Abcam, United States) and DCX (AB2253, Millipore, United States), the double-immunofluorescence staining, was performed to observe neurogenesis, and sections were incubated with primary antibodies overnight at 4°C. After rinsing three times in PBS for 5 min each, sections were incubated for 1 h at 37°C with a secondary antibody. Fluorescence signals were examined under an EVOS M5000 imaging system.

For TUNEL assay, frozen slices were stained with the TUNEL technique using an apoptosis detection kit (MA0223, Meilunbio, China) according to the manufacturer’s protocols. In brief, slices were fixed with 30 min treatment of 4% PFA at room temperature. The liquid was gently aspirated, and the slices were immersed in PBS and incubated at room temperature for 10 min. Furthermore, 100 μl of proteinase K (20 μg/ml) was added to each sample so that the solution covers the entire sample area and incubated for 10 min at room temperature. Then, 50 μl TUNEL detection solution was added dropwise to the sample and incubated in the dark for 60 min at 37°C. After all, the slices were washed 2–3 times with PBS and examined under an EVOS M5000 imaging system.

### Infarct Volume Measurement and MRI

Rats were anesthetized with 1.5–2.5% isoflurane. MR scans were performed 7 and 28 days after rTMS using a 7 Tesla system with Bruker console (Bruker Biospin, PharmaScan 70/16, United States). For T2-weighted imaging, a 35 mm^2^ × 35 mm^2^ field of view (FOV) and a 128 × 128 image matrix were used to obtain the initial RARE anatomical scan, where all six echoes were at equal echo time intervals and under the same readout gradient polarity in T2 × WI. The total time for each sequence was about 5–10 min. Finally, the area of interest of each animal was measured to quantify the ischemic volume.

### Neurobehavioral Evaluation

#### Modified Neurological Severity Score

The neurobehavioral motor outcome was evaluated using the mNSS, performed at 7 and 28 days after rTMS. mNSS includes four tests, namely, motor, sensory, balance, and reflex tests. Scores from all the tests were summed to give the mNSS a score of 0–18. Rats with moderate neurological dysfunction (scores of 7–12) at 2 days after rTMS were selected for use in the subsequent experimental procedures.

#### Novel Object Recognition Tests and Morris Water Maze Test

The novel object recognition (NOR) test and the Morris water maze (MWM) are used to evaluate neurobehavioral cognitive function of each group at 28 days after rTMS as previously described (Luo et al., 2017).

In brief, the NOR test values were expressed as a percentage of the discrimination ratio calculated according to the following formula: Discrimination ratio (%) = (N − F) / (N + F) × 100%, where *N* represents the time spent in exploring the new object and F represents the time spent in exploring the same object.

The rats first received place navigation test for 5 days for MWM. Rats were gently put into the water maze and released facing the wall from one of four quadrants in a random order. They were allowed to find the escape platform for 60 s. The escape latency was measured and analyzed. A 60-s spatial probe test was conducted with the platform removed at the day after the place navigation test. The dwelling time in the target quadrant where the platform was located before and the time of crossing the platform area were recorded during the training.

### Statistical Analysis

All results are expressed as mean ± SEM. Statistical comparisons were made using a Student’s *t*-test (between two groups) or a one-way ANOVA (for multigroup comparisons). *P* < 0.05 was considered to represent a significant difference. Analysis and graphing were performed using the Prism 7.0 software package. Figure 8 was obtained using BioRender.com.

## Results

### Neurological Function Assay

**FIGURE 1:**
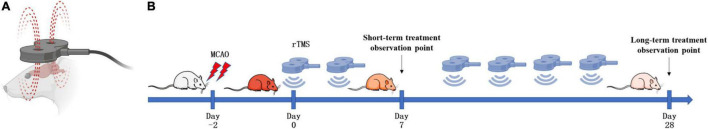
Schematic diagram of the strategy to stimulate ischemic rats. **(A)** Stimulation coil and method. A magnetic stimulator with a figure-of-eight coil was used to stimulate conscious rats. **(B)** Experimental schedule. Rats were treated with repetitive transcranial magnetic stimulation (rTMS) at day 3 after cerebral ischemia.

**FIGURE 2:**
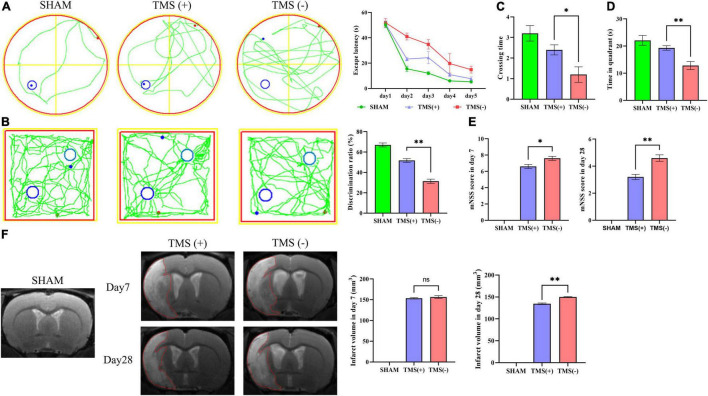
Analysis of neural function recovery of experimental ischemic rats. **(A)** Representative swimming path of each group in the Morris water maze test and analysis of the escape latency to the platform during the training days of the Morris water maze test (*n* = 5 per group). **(B)** Exploration patterns were recorded in the novel object recognition (NOR) test; green lines are exploring patterns of rats; blue circles represent two different objects. Discrimination ratios were measured (*n* = 5 per group). **(C)** Comparisons of the times crossing the target area and **(D)** time spent in the target quadrant during the probe trial (*n* = 5 per group). **(E)** Comparison of modified neurological severity scores (mNSS) in each group (*n* = 5 per group). **(F)** Cerebral infarct volume reflected by MRI and comparison of infarct volume in each group (*n* = 3 per group). Data are shown as mean ± SEM. Statistical significance was determined with the unpaired Student’s *t*-test. *P < 0.05 and **P < 0.01, and ns means no significant.

As shown in Figure 1A, rats were treated with rTMS at 3 days after cerebral ischemia. To investigate the best course of treatment, rTMS were applied to the ischemic rats, and short-term treatment observation point (at day 7) and long-term treatment observation point (at day 28) were used to evaluate the treatment effect (Figure 1B). At 28 days after rTMS, as indicated by the results of NOR and MWM tests, the TMS (+) group shows a significant improvement in neurocognitive behavioral function. The rats were trained on the cued water maze, and escape latency decreased after rTMS treatment (Figure 2A). Compared with TMS (−) group, rats subjected to rTMS exhibited a higher discrimination ratio (Figure 2B). In the spatial probe test, rats subjected to rTMS showed greater numbers of platform crossings (Figure 2C) and longer time spent in the target quadrant (Figure 2D).

To investigate the effects of different course of rTMS treatment on neurological function in ischemic rats, the mNSS scale was applied to assess the neurological function. Compared with TMS (−) group, TMS (+) group exhibited a lower mNSS values at day 7, and the mNSS values became even lower at day 28 (Figure 2E). Besides, the relative infarct volume showed no difference in the TMS (+) group compared to TMS (−) group at 7 days after rTMS, and the TMS (+) group exhibited a reduced infarct volume at day 28 (Figure 2F). The above data proved that rTMS could improve the recovery of neurological function, and long-term treatment presented a better therapeutic effect.

### Neural Regeneration and Tissue Apoptosis

**FIGURE 3:**
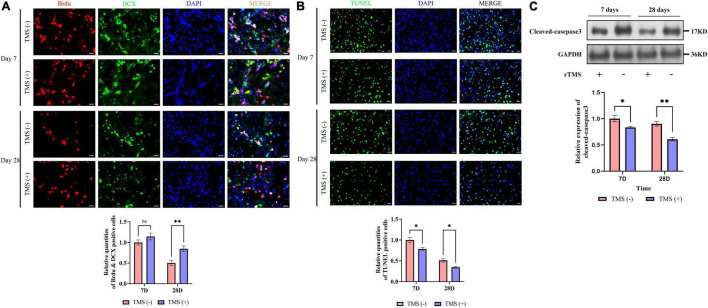
Effects of rTMS on neural regeneration and apoptosis. **(A)** Quantitative analysis of BrdU^+^ and DCX^+^ newborn neural precursor cells (NPCs) in TMS (–) and TMS (+) groups (*n* = 5). Quantity of NPCs in TMS (–) group of day 7 was normalized as control. **(B)** Quantitative analysis of TUNEL-positive apoptotic cells in transcranial magnetic stimulation (TMS) (–) and TMS (+) groups (*n* = 3). Quantity of TUNEL-positive cells in TMS (–) group of day 7 was normalized as control. **(C)** Western blotting analysis of cleaved-caspase3 in TMS (–) and TMS (+) groups (*n* = 4). Expression levels of cleaved-caspase3 in TMS (–) group of day 7 were normalized as control. Data are shown as mean ± SEM. Statistical significance was determined with the unpaired Student’s *t*-test. Scale bar, 10 μm, *P < 0.05 and **P < 0.01, and ns means no significant.

To verify whether rTMS contributes to the neural regeneration, the effect of short-term (7 days) and long-term (28 days) rTMS on BrdU^+^ and DCX^+^ NPCs was analyzed. As shown in Figure 3A, compared with TMS (−) group, the level of NPC population in TMS (+) group was close in day 7, whereas the level in TMS (+) group was higher for the population analysis in day 28. The results suggested that rTMS play an important role in the neural regeneration, and long-term rTMS could better support neurogenesis.

Additionally, we analyzed the potential role of rTMS in apoptosis (Figure 3B). We found rTMS also partially restored the apoptosis of the cerebral cortex in short-term and long-term treatment. Simultaneously, the expression of cleaved-caspase3, a critical executioner of apoptosis, was significantly downregulated in the TMS (+) group of both short-term and long-term treatment (Figure 3C). All the above data suggest that short-term and long-term treatments of rTMS play an important role in reducing cerebral cortex apoptosis.

### Neurotoxic Effects Assay

**FIGURE 4:**
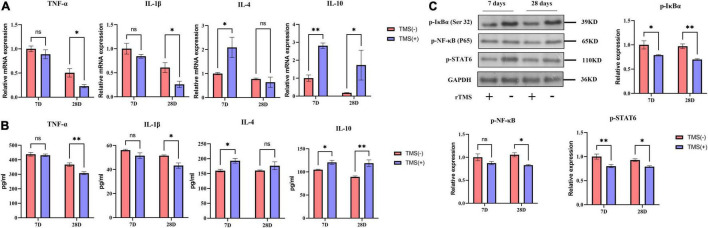
Effects of rTMS on inflammation regulation in experimental ischemic rats. mRNA expression levels **(A)** and secretion **(B)** of inflammatory and anti-inflammatory cytokines in TMS (–) and TMS (+) groups (*n* = 3). **(C)** Normalized expression of phosphorylation of nuclear factor of kappa light polypeptide gene enhancer in B-cells inhibitor, alpha (IκBα), nuclear factor kappa B (NF-κB), and signal transducer and activator of transcription 6 (STAT6) in TMS (–) and TMS (+) groups (*n* = 3). Data are shown as mean ± SEM. Statistical significance was determined with the unpaired Student’s *t*-test. *P < 0.05 and **P < 0.01, and ns means no significant.

Cerebral ischemia rats are known to suffer neurotoxic effects caused by the secretion of proinflammatory cytokines. TNF-α and IL-1β are classical inflammatory cytokines after cerebral ischemia, while IL-4 and IL-10 are anti-inflammatory cytokines. As shown in Figure 4A, compared with the TMS (−) group, the mRNA levels of TNF-α and IL-1β were not obviously affected by rTMS at day 7 but was inhibited by rTMS at day 28. Similar phenomena were also observed through ELISA. As shown in Figure 4B, long-term treatment of rTMS showed a significant inhibition of TNF-α and IL-1β. However, qPCR and ELISA both showed that IL-4 and IL-10 mRNA expression and protein secretion were increased in the rTMS treatment group at day 7, and IL-10 maintained high expression and secretion in the rTMS treatment group at day 28 (Figures 4A,B).

Nuclear factor kappa b (NF-κB) and signal transducer and activator of transcription 6 (STAT6) are transcription factors previously reported to be involved in the secretion of proinflammatory cytokines. Many studies believe that neuroinflammatory damage caused by cerebral ischemia is highly related to their activation. Therefore, we explored whether these factors are involved in the rTMS-mediated regulation of inflammatory factors. After rTMS treatment, the protein levels of p-IκBα, p-NF-κB, and p-STAT6 were significantly downregulated (Figure 4C). These findings indicated that rTMS could inhibit the activation of the NF-κB and STAT6.

### Polarization of Microglia in Ischemic Rats

**FIGURE 5:**
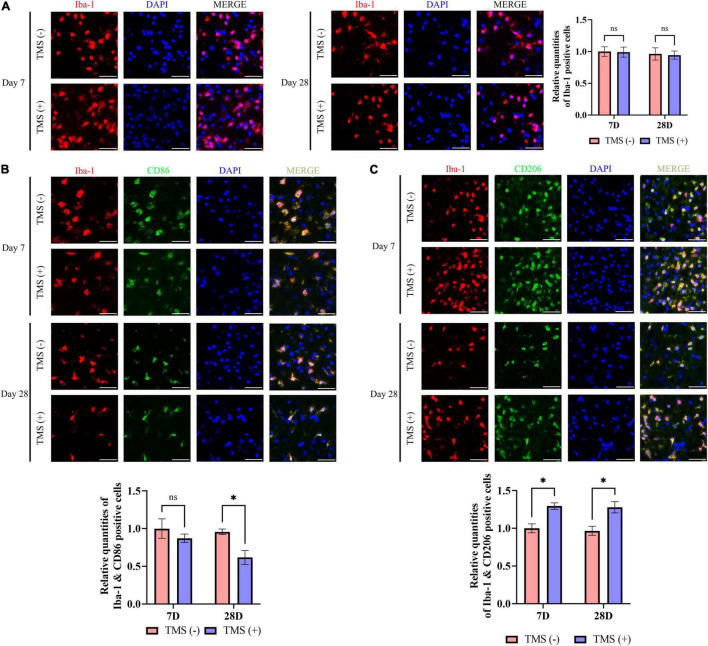
Neuroprotective transformation of microglia in ischemic cerebral cortex. **(A)** Quantitative analysis of Iba-1 positive microglia in TMS (–) and TMS (+) groups (*n* = 3). Quantity of Iba-1 positive cells in TMS (–) group was normalized as control. Iba-1^+^ and CD86^+^ neurotoxic **(B)** and Iba-1^+^ and CD206^+^ neuroprotective **(C)** microglia in TMS (–) and TMS (+) groups (*n* = 3). Quantity of Iba-1^+^ and CD86^+^ or Iba-1^+^ and CD206^+^ cells in TMS (–) group was normalized as control. Data are shown as mean ± SEM. Statistical significance was determined with the unpaired Student’s *t*-test. Scale bar, 20 μm, *P < 0.05, and ns means no significant.

Microglia include an amount of principal immune cells in the brain that respond to the pathophysiological changes induced by IS. As shown in Figure 5A, short-term and long-term treatment of rTMS did not change the percentage of the proliferation rate of microglia. Furthermore, we analyzed the polarization phenotype of microglia and found that rTMS could effectively reduce the inflammatory polarization of Iba-1^+^ and CD86^+^ microglia in long-term rTMS treatment, while there was no obvious change in short-term rTMS treatment group (Figure 5B). When comparing anti-inflammatory polarization of Iba-1^+^ and CD206^+^ microglia in TMS (−) and TMS (+) group, the Iba-1^+^ and CD206^+^ microglia in short-term and long-term rTMS treatment group were both increased (Figure 5C). The above data indicated that rTMS might promote anti-inflammatory transformation of microglia in ischemic rats.

**FIGURE 6:**
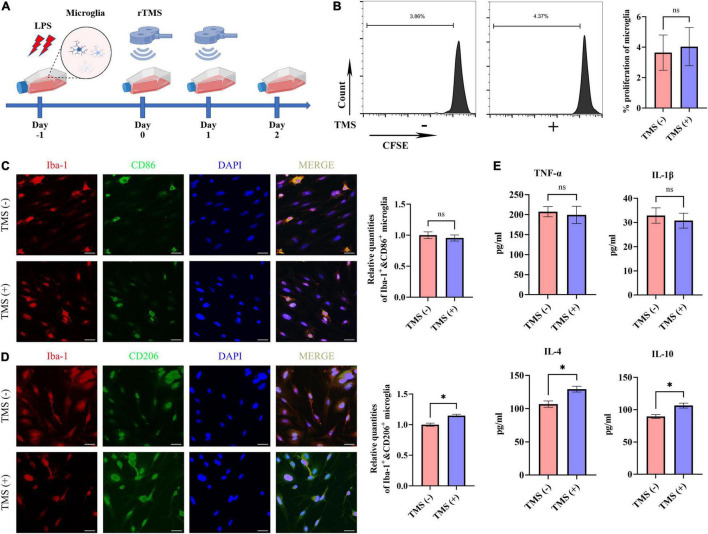
Neuroprotective polarization of microglia activated by lipopolysaccharide (LPS). **(A)** The experiment timelines of rTMS stimulation for microglia activated by LPS. Microglia were treated with rTMS at 1 day after LPS stimulation. **(B)** Carboxyfluorescein succinimidyl ester (CFSE) analysis of cell proliferation. Quantitative analysis of Iba-1^+^ and CD86^+^ neurotoxic microglia **(C)** and Iba-1^+^ and CD206^+^ neuroprotective microglia **(D)** in TMS (–) and TMS (+) groups (*n* = 3). Quantity of Iba-1^+^ and CD86^+^ or Iba-1^+^ and CD206^+^ cells in TMS (–) group was normalized as control. **(E)** The secretion of inflammatory and anti-inflammatory cytokines in TMS (–) and TMS (+) groups (*n* = 3). Data are shown as mean ± SEM. Scale bar, 10 μm, *P < 0.05 and **P < 0.01, and ns means no significant.

Lipopolysaccharide has been reported to induce the proinflammatory transition of microglia. Hence, to further verify whether rTMS could contribute to the polarization transition of microglia, PM were extracted, and rTMS were applied to PM activated by LPS *in vitro* (Figure 6A). As shown in Figure 6B, PM exhibited low proliferative activity *in vitro* with or without rTMS treatment, for there was nearly no obvious change in the population of proliferation cell. Then, the effects of rTMS on microglia polarization were further verified. With rTMS treatment, PM showed no obvious change tendency of CD86^+^ phenotype but an obvious increase in CD206^+^ cell proportion (Figures 6C,D).

To further confirm the rTMS effects reverse neurotoxic microglia into neuroprotective phenotype *in vitro*, we then analyzed the secretion of proinflammatory cytokines (TNF-α and IL-1β) and anti-inflammatory cytokines (IL-4 and IL-10) in PM. In comparison with the TMS (−) group, there was no obvious difference in the secretion of proinflammatory cytokines TNF-α and IL-1β (Figure 6E), whereas the secretion of anti-inflammatory cytokines IL-4 and IL-10 was upregulated in the TMS (+) group (Figure 6E). Overall, these results indicated that rTMS could promote the anti-inflammatory polarization and anti-inflammatory cytokine secretion of microglia.

### Neural Stem Cells Treated With Conditioned Medium From Microglia

**FIGURE 7:**
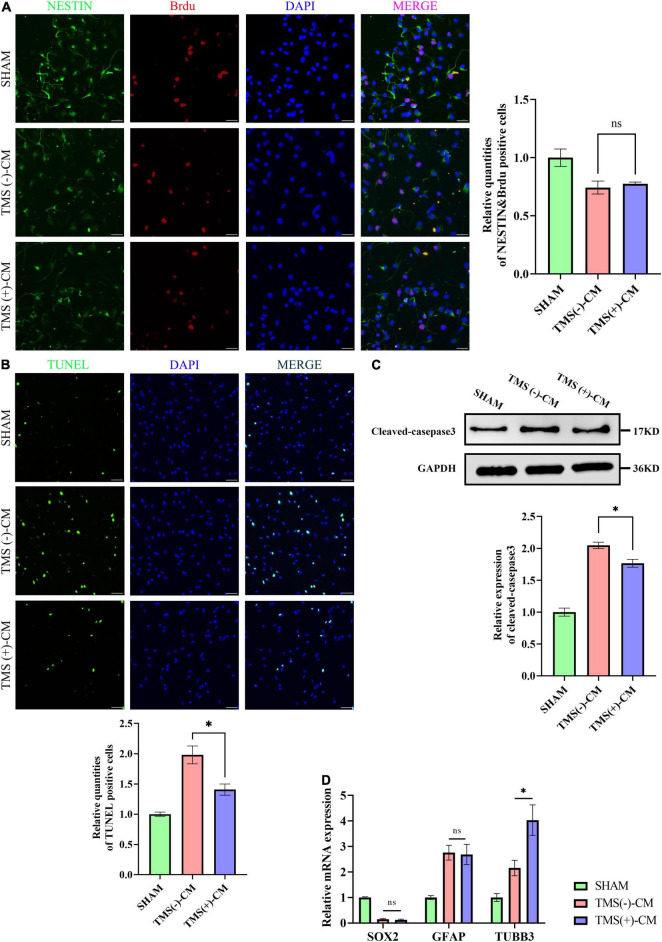
Effect of microglia-derived conditioned medium (CM) on neural stem cells (NSCs). **(A)** Quantitative analysis of BrdU^+^ and NESTIN^+^ NSCs in NSCs treated with or without microglia-derived CMs (*n* = 3), scale bar, 10 μm. Quantity of NESTIN^+^ and BrdU^+^ cells in TMS (–) group was normalized as control. **(B)** Quantitative analysis of TUNEL-positive apoptotic cells in NSCs treated with or without microglia-derived CMs (*n* = 3), Scale bar, 25 μm. Quantity of TUNEL-positive cells in TMS (–) group was normalized as control. **(C)** Western blotting analysis of cleaved-caspase3 in NSCs treated with or without microglia-derived CMs (*n* = 3). **(D)** Relative mRNA expression of SOX2, GFAP, and TUBB3 (*n* = 3). Data are shown as mean ± SEM. Statistical significance was determined with the unpaired Student’s *t*-test. *P < 0.05, and ns means no significant.

Neural stem cells were isolated and highly expressed NESTIN and SOX2. To investigate the effect of different microglial CM on NSCs characteristics, NSCs were cultured in CM collected from non-rTMS-stimulated microglia [TMS (−)-CM] or rTMS-stimulated microglia [TMS (+)-CM]. NSCs cultured in TMS (−)-CM or TMS (+)-CM had a lower quantities of BrdU incorporation cells, whereas TMS (+)-CM group showed no obvious difference from TMS (−)-CM group (Figure 7A). Moreover, when we looked at the effect of apoptosis by CM culture, TMS (−)-CM and TMS (+)-CM both increase the apoptosis rate of NSCs. However, the TUNEL incorporation of TMS (+)-CM was relative less than TMS (−)-CM group (Figure 7B), and the expression of cleaved-caspase3 was lower (Figure 7C).

Additionally, we further tested whether CM could regulate the NSCs fate. The results indicated that both TMS (−)-CM and TMS (+)-CM could promote NSCs differentiation for the expression of stem cell marker SOX2 was obviously downregulated (Figure 7D). In addition, TMS (−)-CM and TMS (+)-CM both displayed an obvious stimulation effect to upregulate the expression of astrocytes marker GFAP, whereas TMS (+)-CM showed a stronger effect to upregulate the expression of neuronal precursor cell marker TUBB3 (Figure 7D). The results indicate that rTMS can promote neuronal differentiation through affecting microglia polarization.

## Discussion

In the past few years, the general safety and efficacy of rTMS for the functional recovery after IS have been demonstrated in numerous basic research and clinical trials (Luber and Lisanby, 2014; Liu et al., 2020; Wang et al., 2020; Yin et al., 2020; Hong et al., 2021; Natale et al., 2021). This study confirms that the function of rTMS in relieving nerve inflammation damage and promotes nerve tissue regeneration are related to promoting the anti-inflammatory polarization of microglia. rTMS could improve functional recovery, which is associated with the reduction of apoptosis and enhancement of neurogenesis. Results *in vivo* and *in vitro* analysis indicated that rTMS could promote the anti-inflammatory polarization of microglia in an independent way. Collectively, these characteristics revealed that the anti-inflammatory polarization of microglia could secrete more neuroprotective anti-inflammatory cytokines for the healing of inflammatory injury. Further testing in comparison between short-term and long-term treatment of rTMS confirmed that long-term rTMS could obtain better nerve repair and inflammation control effect. We concluded that rTMS shows a significant transition of anti-inflammatory polarization of microglia for treating IS.

Inflammation is initiated during the acute phase of stroke and is likely to become the predominant injury mechanism lasting for several days. Generally speaking, proinflammatory intracellular signaling cascades lead to the release of proinflammatory cytokines, such as IL-1β and TNF-α, to initiate a local immune response. This process results in local and systemic immune system activation, that exacerbates neuronal injury. However, it is also reported that inflammatory may contribute to neuroprotection and later tissue repair (Jayaraj et al., 2019; Lambertsen et al., 2019). Therefore, it is a tricky choice to not only relieve stroke but also reduce inflammation. From the perspective of safety and effectiveness, the goal of recent experiments seems to have shifted from regulating of proinflammatory mechanisms to promoting a more protective cell phenotype, with the goal of promoting neural recovery. In fact, our data indicated that rTMS did not seem to be a way to quickly eliminate inflammation, for IL-1β and TNF-α were not reduced in a short time. However, IL-4 and IL-10 were quickly upregulated and rTMS maintained their high-expression for a long time. It not only ensures the long-term control of inflammation but also facilitates the repair of nerves under inflammation.

Microglia are important players of the innate immune system and the first responders to the ischemic tissue. It has been well established that the effect of microglia is considered with neuroprotective and neurotoxic aspects. Activated neurotoxic microglia could secrete proinflammatory cytokines (e.g., IL-1β and TNF-α) and further enlarge the infarct. Conversely, neuroprotective microglia could secrete anti-inflammatory cytokines (e.g., IL-4 and IL-10) and protect neurons against ischemic damage. Previous studies have revealed that rTMS could induce a shift in microglia phenotype activation *in vivo* and reduce proinflammatory cytokines (Hong et al., 2020; Zong et al., 2020). Our data are partially similar to the above research conclusions, for rTMS could promote the anti-inflammatory polarization of microglia. In our study, we also found that rTMS would not change the total number or proportion of microglia *in vivo* or *in vitro* treatment. In addition, we did not find rTMS could downregulate the proportion of proinflammatory CD86^+^ microglia *in vitro*, and same phenomenon existed *in vivo*. Although long-term rTMS treatment showed the ability to reduce the expression of CD86, it might be caused by promoting other functional cells such as astrocytes to secrete anti-inflammatory factors to act on the neurotoxic microglia. Therefore, our data are more inclined to believe that rTMS can promote the polarization of anti-inflammatory microglia to exert long-term neuroprotective functions, rather than directly change proinflammatory polarization of microglia.

Microglia are believed to affect the NSCs fate. Enhanced astrocytogenesis and neurogenesis were observed, while NSCs were treated with CM from microglia stimulated with LPS (Nakanishi et al., 2007; Shigemoto-Mogami et al., 2014). Our results showed that both TMS (−)-CM and TMS (+)-CM could promote NSCs differentiation. Compared with TMS (−)-CM, TMS (+)-CM exhibited a higher tendency to differentiate into neurons, whereas TMS (−)-CM were more likely to induce NSCs into astrocytes. Differences in soluble cytokine types of CMs may explain the differences in NSCs fate. For example, IL-1β is believed to reduce NSC or NPC proliferation, and neuronal differentiation (Crampton et al., 2012; Zunszain et al., 2012; Chen et al., 2013), but enhance astrogliogenesis (Ling et al., 1998). Kynurenine pathway and signal transducer and activator of transcription 3 (STAT3) pathway are believed to be activated by IL-1β and contribute to the above effect (Zunszain et al., 2012; Chen et al., 2013); TNF-α decreases neurogenesis and upregulates the expression of HES-1, previously believed as an antineurogenic transcription (Nakamura et al., 2000; Johansson et al., 2008); IL-4 and IL-10 both have antiapoptotic effect. IL-4 can reduce the proliferation rate but promote neuronal and glial differentiation, while IL-10 promotes the proliferation but its effect on differentiation is not obvious (Kiyota et al., 2012; Lim et al., 2013). The soluble cytokines from microglia have both positive and negative role on neurogenesis or neuroapoptosis, while some researchers have recently explained the effects of rTMS on brain from other perspective of regulating the neurogenesis and dendritic complexity in the hippocampus or primary motor cortex (Cambiaghi et al., 2021, 2020). High-frequency rTMS can affect the type and quantity of the cytokines by regulating the polarization of microglia, which partly explains the role of rTMS in promoting neurogenesis and inhibiting neuroapoptosis *in vivo* and *in vitro*.

## Conclusion

**FIGURE 8:**
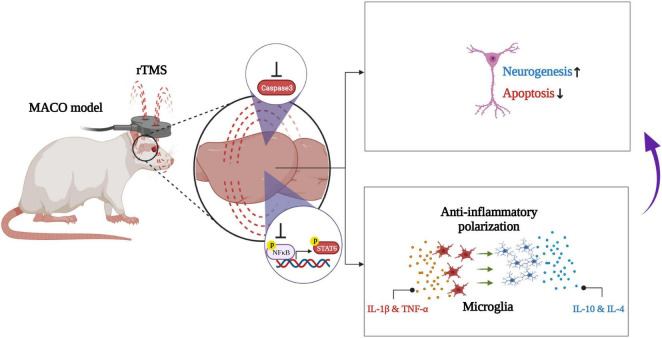
Schematic illustration of rTMS-mediated anti-inflammatory polarization of microglia and neural recovery.

In conclusion, our work demonstrates that rTMS helps neurorehabilitation by promoting the anti-inflammatory polarization of microglia (Figure 8). These data preliminarily confirm that rTMS can independently induce the neuroprotective phenotype of microglia. Based on our findings, we suggested that rTMS can further explore the optimization of treatment parameters in response to changes in microglial phenotype.

## Data Availability Statement

The original contributions presented in the study are included in the article/Supplementary Material, further inquiries can be directed to the corresponding authors.

## Ethics Statement

The animal study was reviewed and approved by the Sun Yat-sen University Institutional Animal Care and Use Committee.

## Author Contributions

XH and FQ were responsible for the study design and manuscript drafting. JL, YF, and ML carried out most of the experimental work, such as MCAO model and immunostaining assay, cell extraction and culturing, Western blot, RNA purification, and real-time-PCR. MY conducted TMS stimulation and data analysis. All authors read and approved the manuscript.

## Conflict of Interest

The authors declare that the research was conducted in the absence of any commercial or financial relationships that could be construed as a potential conflict of interest.

## Publisher’s Note

All claims expressed in this article are solely those of the authors and do not necessarily represent those of their affiliated organizations, or those of the publisher, the editors and the reviewers. Any product that may be evaluated in this article, or claim that may be made by its manufacturer, is not guaranteed or endorsed by the publisher.
