# Supplementary material for: Repetitive Transcranial Magnetic Stimulation Improves Neurological Function and Promotes the Anti-inflammatory Polarization of Microglia in Ischemic Rats
Source: Front Cell Neurosci. 2022 Apr 12;16:878345. doi: 10.3389/fncel.2022.878345 (PMC9039226; doi:10.3389/fncel.2022.878345)
Supplement: Supplementary file 1 [file Table_1.DOCX]

**Table S1. Primers used for the amplification of transcripts by qPCR**

| **Genes** | **Forward sequence** | **Reverse sequence** |
| --- | --- | --- |
| GAPDH | 5’-GGCAAGTTCAACGGCACAGTCA-3’ | 5’-CGACATACTCAGCACCAGCATCAC -3’ |
| TNF-a | 5’-TgCCTCAgCCTCTTCTCATTCC-3’ | 5’-GGGCAGCCTTGTCCCTTGAA -3’ |
| IL-1β | 5’-CCTCTGTGACTCGTGGGATGATGA-3’ | 5’-TTTGTCGTTGCTTGTCTCTCCTTGT-3’ |
| IL-4 | 5’-AGACAGCCACTCACCTCTTCAG-3’ | 5’-TTCTGCCAGTGCCTCTTTGCTG-3’ |
| IL-10 | 5’-TCTCCGAGATGCCTTCAGCAGA-3’ | 5’-TCAGACAAGGCTTGGCAACCCA-3’ |
| SOX2 | 5’-CGCTTCGCAGGGAGTTCTCAAA-3’ | 5’-TCCTTCCTTGTCTGTAACGGTCCTT-3’ |
| GFAP | 5’-CGGTGGAGATAACTTGGACAATGGA-3’ | 5’-CTCAGAAGGATGGTTGTGGACTCTT-3’ |
| TUBB3 | 5’-ATGAAGGAGGTGGACGAGCAGATG-3’ | 5’-GCTGTGCTATTGCCGATGAAGGT-3’ |
